# ApoE4 induces Aβ42, tau, and neuronal pathology in the hippocampus of young targeted replacement apoE4 mice

**DOI:** 10.1186/1750-1326-8-16

**Published:** 2013-05-17

**Authors:** Ori Liraz, Anat Boehm-Cagan, Daniel M Michaelson

**Affiliations:** 1 Department of Neurobiology, The George S. Wise Faculty of Life Sciences, The Sagol School of Neuroscience, Tel Aviv University, Ramat Aviv, 69978 Tel Aviv, Israel

## Abstract

**Background:**

Recent findings suggest that the pathological effects of apoE4, the most prevalent genetic risk factor for Alzheimer’s disease (AD), start many years before the onset of the disease and are already detectable at a young age. In the present study we investigated the extent to which such pathological and cognitive impairments also occur in young apoE4 mice.

**Results:**

This study revealed that the levels of the presynaptic glutamatergic vesicular transporter, VGlut, in the CA3, CA1, and DG hippocampal subfields were lower in hippocampal neurons of young (4-month-old) apoE4-targeted replacement mice than in those of the apoE3 mice. In contrast, the corresponding inhibitory GABAergic nerve terminals and perikarya were not affected by apoE4.

This synaptic effect was associated with hyperphosphorylation of tau in these neurons. In addition, apoE4 increased the accumulation of neuronal Aβ42 and induced mitochondrial changes, both of which were specifically pronounced in CA3 neurons. Spatial navigation behavioral studies revealed that these hippocampal pathological effects of apoE4 are associated with corresponding behavioral impairments. Time-course studies revealed that the effects of apoE4 on tau hyperphosphorylation and the mitochondria were already apparent at the age of 1 month and that the apoE4-driven accumulation of neuronal Aβ and reduced VGlut levels evolve later and are apparent at the age of 2–4 months. Furthermore, the levels of tau phosphorylation decrease in apoE3 mice and increase in apoE4 mice between 1 and 4 months, whereas the levels of Aβ42 decrease in apoE3 mice and are not affected in apoE4 mice over the same time period.

**Conclusions:**

These findings show that apoE4 stimulates the accumulation of Aβ42 and hyperphosphorylated tau and reduces the levels of VGlut in hippocampal neurons of young apoE4-targeted replacement mice and that these neurochemical effects are associated with cognitive impairments. This model is not associated with hypothesis-driven mechanistic manipulations and is thus most suitable for unbiased studies of the mechanisms underlying the pathological effects of apoE4.

## Introduction

Alzheimer’s disease (AD), the most prevalent form of dementia in the elderly, is characterized by cognitive decline and by the occurrence of brain senile plaques and neurofibrillary tangles (NFT) as well as by the loss of brain synapses and neurons [1-3]. The senile plaques contain a 40-42-amino acid-long amyloid-beta (Aβ) peptide derived from a precursor protein (APP) [3,4]. Aβ is also present in the brain as soluble oligomers, which play an important and early role in neurodegeneration in AD [5-8]. The NFT contain abnormal aggregates of the microtubule-associated protein, tau, which leads to disruption of the neuronal cytoskeleton followed by neurodegeneration and cell death [9,10]. Several chemical modifications have been described in NFT's tau, of which hyperphosphorylation is a key event [11,12]. The classical neuropathological studies of Braak & Braak revealed that the AD lesions begin to form 20–30 years before the disease becomes clinically evident [13]. This has now been corroborated by longitudinal imaging studies, which revealed that brain atrophy and Aβ deposition begin during the preclinical stage of the disease [14-17]. Synaptic dysfunction and loss is the earliest histological neuronal pathology in AD and is associated with early loss of dendritic spines and with presynaptic and postsynaptic impairments [18,19], which correlate with cognitive decline at the early stages of the disease [20]. The synaptic pathology is particularly pronounced in distinct brain areas such as the hippocampus.

Genetic studies revealed allelic segregation of the apolipoprotein E (apoE) gene to families with a higher risk of late-onset AD and of sporadic AD [21-23]. There are three major alleles of apoE, termed E2 (apoE2), E3 (apoE3), and E4 (apoE4), of which apoE4 is the AD risk factor. The frequency of apoE4 in sporadic AD is >50%, and it increases the risk for AD by lowering the age of onset of the disease by 7 to 9 years per allele copy [22]. Pathologically, apoE4 is associated with increased deposition of Aβ [24,25], hyperphosphorylation of tau [26,27], as well as impaired neuronal plasticity and neuropathology [28,29]. Declining memory and brain pathology have been reported in middle-aged apoE4 carriers with an ongoing normal clinical status [30,31], suggesting that the effects of apoE4 begin decades before the onset of AD.

The finding that Aβ deposition is specifically elevated in apoE4-positive AD patients [25], combined with *in vivo* and *in vitro* model studies, which revealed that apoE4 and the amyloid cascade interact synergistically [12,32-36], led to the suggestion that the pathological effects of apoE4 are mediated via cross-talk with the amyloid cascade [37-40]. The central role of apoE in the transport and delivery of brain lipids and the finding that the binding of apoE to lipoproteins is affected by the apoE genotype [41] led to the proposal that the pathological effects of apoE4 are mediated via lipid-related mechanisms, possibly through the effects of lipids on neural and synaptic function and morphology. ApoE is expressed in stressed and injured neurons [42] and transgenic over-expression of apoE4 in neurons increases tau phosphorylation [43,44]. This led to an additional hypothesis, namely, that the pathological effects of apoE4 are mediated by intraneuronal Aβ and stimulation of tau hyperphosphorylation [43].

Accumulating evidence suggest that mitochondrial dysfunction occurs early in AD and plays a key role in the disease [45]. *In vivo* and *in vitro* model studies revealed that the pathological effects of apoE4 are associated with enhanced mitochondrial pathology, such as decreased activity of mitochondrial enzymes, specifically, cytochrome C oxidase (COX) [46,47]. Recent studies suggest that regions within the gene coding for the translocase of the outer mitochondrial membrane, Tom40, and the apoE gene interact genetically and share common enhancers [48]. Taken together, these findings suggest that the mitochondria are an early and important intracellular target of apoE4.

The existence of several suggested mechanisms has important implications regarding the design and use of appropriate apoE4-related *in vivo* models. Accordingly, models such as APP and apoE4 double transgenic mice [49] and pharmacological activation of the amyloid cascade in apoE4 mice [32,33] are most suitable for assessing the role of cross talk interactions between apoE4 and the amyloid cascade, whereas mice in which apoE4 is expressed preferentially in neurons [50] are suitable for studying the pathological consequences of intraneuronal apoE4 and its catabolites and their interactions with tau. In view of the numerous apoE4-related mechanistic hypotheses, it is important to develop and employ “mechanistically unbiased” models in which the pathological effects of apoE4 are not triggered by exposure to a theory and a mechanistic hypothesis-driven paradigm. Since the pathological effects of apoE4 in humans begin many years before the onset of the disease and are already detectable at a young age, a possible application of this hypothesis-independent approach is to focus on the early effects of apoE4.

In the present study we adopted this approach utilizing young 4-month-old targeted replacement mice free of any exterior manipulations. In view of the documented presynaptic and mitochondria-related effects of apoE4 and the cross talk between apoE4 and tau [32,33,49,50], the study focuses on these parameters and on assessing the extent to which these effects are associated with cognitive impairments and the age at which they evolve.

## Materials and methods

### Transgenic mice

ApoE-target replacement mice, in which the endogenous mouse apoE was replaced by either human apoE3 or apoE4, were created by gene targeting, as previously described [51]. The mice used were purchased from Taconic (Germantown, NY). Mice were back-crossed to wild-type C57BL/6J mice (Harlan 2BL/610) for ten generations and were homozygous for the apoE3 (3/3) or apoE4 (4/4) alleles. These mice are referred to in the text as apoE3 and apoE4 mice, respectively. The apoE genotype of the mice was confirmed by PCR analysis, as described previously [33,52]. All the experiments were performed on age-matched male animals (1 to 4 months of age), and were approved by the Tel Aviv University Animal Care Committee. Every effort was made to reduce animal stress and to minimize animal usage.

### Immunohistochemistry and immunofluorescence confocal microscopy

Mice were anesthetized with ketamine and xylazine and perfused transcardially with saline and then with 4% paraformaldehyde in 0.1M phosphate buffer, pH 7.4. Their brains were removed, fixed overnight in 4% paraformaldehyde in 0.1 M phosphate buffer, pH 7.4, and then placed in 30% sucrose for 48 h. Frozen coronal sections (30 μm) were then cut on a sliding microtome, collected serially, placed in 200 μl of cryoprotectant, and stored at −20°C until use. The free-floating sections were immunostained with the following primary antibodies (Abs): Rabbit anti-Aβ42 (1:500; Chemicon, Temecula, CA); Rabbit anti-Aβ40 (1:500; Chemicon); Mouse anti-pan Aβ (4G8, 1:100; Signet); Mouse anti-N-terminal APP (22C11, 1:2000, Millipore); Rabbit anti-tau (H150 ,1:600, Santa Cruz Biotechnology); Rabbit anti-202/205 phosphorylated tau (AT8 ,1:200, Innogenetics); Mouse anti-212/214 phosphorylated tau (AT100 ,1:200, Innogenetics); Rabbit anti-Tom40 (1:500; Santa Cruz); Goat anti-COX1 (1:400; Santa Cruz); Guinea-pig anti-VGlut1 (1:2000; Millipore); Mouse anti-GAD67 (1:250; Millipore); Mouse anti-Vgat (1:200; Synaptic Systems); Mouse anti-Synaptophysin (1:200; Sigma); Mouse anti-NeuN (1:500; Chemicon); Goat anti-apoE (1:5000, Chemicon) and Mouse anti-GFAP (1:2000, Pharmingen).

Immunohistochemistry was performed as previously described [32]. Accordingly, sections were washed with 10 mM PBS, pH 7.4, and blocked for 1 h in 20% serum diluted in PBS with 0.1% Triton X-100 (PBST), after which the primary antibody, diluted in PBST containing 2% of the appropriate serum, was applied overnight at 4°C. The sections were then rinsed in PBST, and incubated for 1 h at room temperature with the corresponding secondary antibody (Vector Laboratories, Burlingame, CA) diluted 1:200 in PBST containing 2% of the appropriate serum. After several additional rinses in PBST, the sections were incubated for 0.5 h in avidin-biotin-horseradish peroxidase complex (ABC Elite; Vector Laboratories) in PBST. After rinses in PBST, sections were placed for up to 10 min in diaminobenzidine chromagen solution (Vector Laboratories). To minimize variability, sections from all animals were stained simultaneously. The reaction was monitored visually and stopped by rinses in PBS. The sections were mounted on a dry gelatin-coated slide and then dehydrated and sealed with cover slips. Aβ staining was performed similarly except that the sections were preincubated with 70% formic acid for 7 min in order to increase antigen retrieval prior to staining. The immunostained sections were viewed using a Zeiss light microscope (Axioskop, Oberkochen, Germany) interfaced with a CCD video camera (Kodak Megaplus, Rochester, NY, USA). Pictures of stained brains were obtained at X10 magnification. Analysis and quantification of the staining (2 hippocampal images per animal at Bregma (−1.7)-(−2.06)) were performed using the Image-Pro plus system for image analysis (v. 5.1, Media Cybernetics, Silver Spring, MD, USA). The images were analyzed by marking the area of interest (e.g., a hippocampal subfield such as CA3) and setting a threshold for all sections of a specific labeling. The stained area above the threshold relative to the total area was then determined for each section. All the groups were stained together and the results presented represent the mean ± SEM of the percent area stained normalized relative to the young apoE3 mice.

Immunofluorescence staining was performed using fluorescent chromogens. Accordingly, sections were first blocked (incubation with 20% normal donkey serum in PBST for 1 h at room temperature), and then reacted for 48 h at 4°C with the primary antibodies (dissolved in 2% normal donkey serum in PBST). Next, the bound primary antibodies were visualized by incubating the sections for 1 h at room temperature with Alexa-fluor 488-conjugated donkey anti-rabbit (1:1000; Invitrogen, Eugene, OR), Alexa-fluor 488-conjugated donkey anti-mouse (1:1000; Invitrogen), or Alexa-fluor 488-conjugated goat anti-Guinea-pig (1:1000; Invitrogen), depending on the appropriate initial antibody. The sections were then mounted on dry gelatin-coated slides. Sections stained for immunofluorescence were visualized using a confocal scanning laser microscope (Zeiss, LSM 510). Images (1024×1024 pixels, 12 bit) were acquired by averaging eight scans. Control experiments revealed no staining in sections lacking the first antibody. The intensities of immunofluorescence staining, expressed as the percentage of the area stained, were calculated utilizing the Image-Pro Plus system (version 5.1, Media Cybernetics) as previously described [32]. All images for each immunostaining were obtained under identical conditions, and their quantitative analyses were performed with no further handling. Moderate adjustments for contrast and brightness were performed evenly on all the presented images of the different mouse groups. The images were analyzed by setting a threshold for all sections of a specific labeling. The area of the staining over the threshold compared to the total area of interest was determined for each mouse and each group was averaged. For the apoE, GFAP and NeuN triple labeling colocalization experiments, each image was first analyzed separately. The colocalizations of apoE with NeuN and of apoE with GFAP were then determined as the percentage of the co-stained area relative to the staining of each of the individual stainings.

### Immunoblot analysis

Immunoblot analysis was performed as previously described [53,54]. In brief, mice were decapitated and their brains were rapidly excised and frozen in liquid nitrogen. The frozen brains were then cut into 500-μm coronal slices utilizing a frozen mold, after which the whole hippocampi or its corresponding CA3 subfield were excised while frozen and stored at −70°C until use. The dissected hippocampus and CA3 samples of each brain were then homogenized in 200 μl or 50 μl, respectively, in the following detergent-free homogenization buffer [10 mM HEPES, 2 mM EDTA, 2 mM EGTA, 0.5 mM DTT, protease inhibitor cocktail (Sigma P8340) and phosphatase inhibitor cocktail (Sigma P5726)]. The homogenates were then aliquoted and stored at −70°C. Gel electrophoresis and immunoblot assays were performed on SDS-treated samples (boiling for 10 min in 0.5% SDS) as previously described [32,53] utilizing the following antibodies: Mouse anti-VGlut1 (1:1000; Millipore), Rabbit anti-Tom40 (1:1000; Santa Cruz), Mouse anti-COX1 (1:1000; Santa Cruz), and Goat anti-apoE (1:10000, Chemicon). Protein concentration was determined utilizing the BCA protein assay kit (Pierce 23225).

The immunoblot bands were visualized utilizing the ECL chemiluminescent substrate (Pierce), after which their intensity was quantified using EZQuantGel software (EZQuant, Tel Aviv, Israel). GAPDH levels were employed as gel loading controls and the results are presented relative to the apoE3 mice.

### Aβ42 ELISA

The levels of mouse Aβ X-42 (Aβ42) were determined utilizing the Beta Amyloid X-42 ELISA kit from Covance (Cat# SIG-38954) according to the manufacturer's specifications. Specifically, whole hippocampi were homogenized in 180 μl Tris buffered saline (20 mM Tris, pH 7.4, containing 150 mM NaCl; TBS) with protease inhibitor (Roche). Triton X-100 was then added to a final concentration of 1% and the samples were agitated by pipetting up and down.

### Behavioral experiments

The spatial navigation test was performed by a dry maze modification of the hole board test [32,55], which monitors the ability of the mice to locate a small water-filled well in a circular arena. The mice were water deprived for 2 days before the experiment, whereas throughout the entire experiment they were subjected to a 23 h per day water deprivation regime, in which they were able to drink ad libium for 1 h every day after being tested. After 2 days of water deprivation, the mice were placed in a circular arena (95 cm diameter, with 20 evenly separated wells; 1 cm depth, 0.5 cm diameter) in which all the wells were filled with 100 μl of water. This was performed 4 times per day for 2 days. Each such run lasted 120 sec, during which the mice were allowed to drink from all the wells that they located during these runs. The arena was cleaned with 70% ethanol between each run. Following this habituation, the mice were placed in the arena, in which only 1 well contained water (4 runs a day, each lasting up to 120 sec). If the mouse found the water-filled well, it was allowed to drink for 15 sec; if the mouse did not find the well, it was brought to it after 120 sec and allowed to remain there for 15 sec. The time required for the mice to reach the well (latency) was measured in seconds. This was performed for 8 days. To elevate the level of complexity of the test, the location of the water-filled well was changed to a novel location on day 9, and the performance of the mice was tested for 5 more days in this configuration. Latency to the water-filled well was measured for each trial.

### Statistical analysis

**Figure 1:**
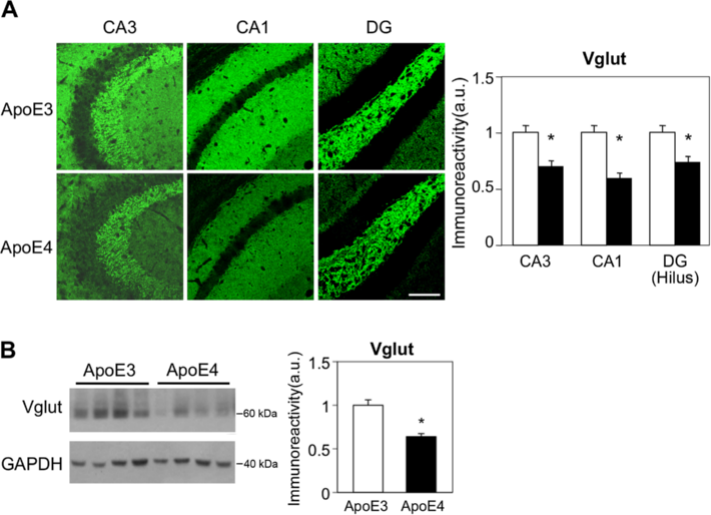
**The levels of the presynaptic gutamatergic transporter VGlut in hippocampal neurons of 4-month-old apoE3 and apoE4 mice. (A)** VGlut1 immunohistochemistry. Representative images (X20 magnification) of the indicated hippocampal subfields are presented on the left. Quantification of the results (mean ± SEM: n=20 per group) of apoE3 mice (white bars) and apoE4 mice (black bars) was performed by computerized image analysis as described in Materials and Methods and is shown on the right. *p<0.001 (for comparison of the results of the apoE4 and apoE3 mice). Scale = 120 μ. **(B)** VGlut immunoblot. Representative immunoblots of homogenates of whole hippocampi of apoE3 and apoE4 mice are presented on the left together with the GAPDH standard. Quantification of the results (mean ± SEM; n=10 per group) of apoE3 mice (white bars) and apoE4 mice (black bars) is depicted on the right. *p<0.001.

**Figure 2:**
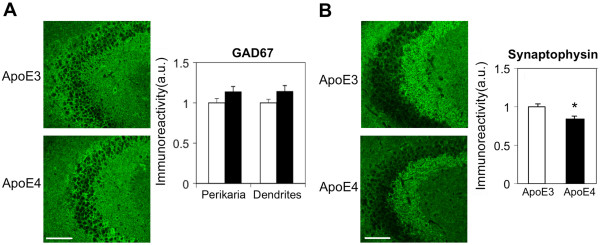
**The levels of inhibitory neurons and synapses (GAD67) and of the presynaptic marker synaptophysin in 4-month-old apoE3 and apoE4 mice. (A)** GAD67 immunohistochemistry. Representative images (X20 magnification) of the CA3 subfield are presented on the left. Quantification of the results in the corresponding perikarya and dendritic fields (mean ± SEM; n=20 per group) of apoE3 mice (white bars) and apoE4 mice (black bars) is shown on the right. Scale = 120 μ. **(B)** Synaptophysin immunohistochemistry. Representative images (X20 magnification) of the CA3 subfield are presented on the left. Quantification of the results (mean ± SEM; n=20 per group) of apoE3 mice (white bars) and apoE4 mice (black bars) is shown on the right. Quantification of the GAD67 and synaptophysin immunohistochemical results was performed by computerized image analysis, as described in Materials and Methods. *p<0.005. Scale = 120 μ.

**Figure 3:**
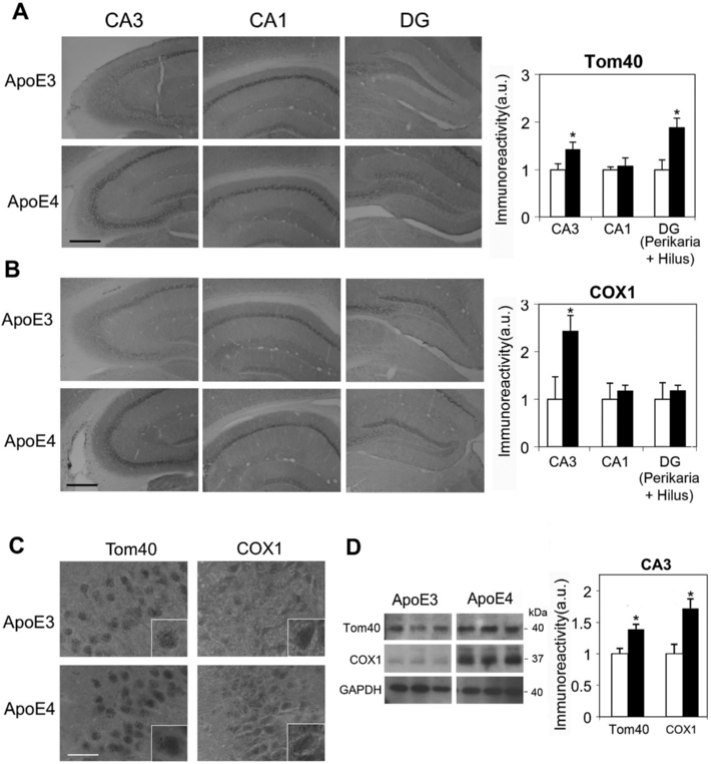
**The levels of the mitochondrial proteins Tom40 and COX1 in hippocampal neurons of 4-month-old apoE3 and apoE4 mice. (A)** Tom40 immunohistochemistry. Representative images (X10 magnification) of the indicated hippocampal subfields are presented on the left. Quantification of the results (mean ± SEM; n=8 per group) of apoE3 mice (white bars) and apoE4 mice (black bars) is shown on the right. *p<0.05. Scale = 350 μ. **(B)** COX1 immunohistochemistry. Representative images (X10 magnification) of the indicated hippocampal subfields are presented on the left. Quantification of the results (mean ± SEM; n=8 per group) of apoE3 mice (white bars) and apoE4 mice (black bars) is shown on the right. *p<0.05. Scale = 350 μ. Quantification of the Tom40 and COX1 results was performed by computerized image analysis, as described in Materials and Methods. **(C)** Representative images of CA3 at X100 magnification are shown. Scale = 30 μ. Inserts depict selected neurons at X2 magnification. **(D)** Tom40 and COX1 immunoblots. Representative immunoblots of homogenates of hippocampal CA3 neurons of apoE3 and apoE4 mice are presented on the left together with the GAPDH standard. Quantification of the results (mean ± SEM; n=5 per group) of apoE3 mice (white bars) and apoE4 mice (black bars) is depicted on the right. *p<0.05.

**Figure 4:**
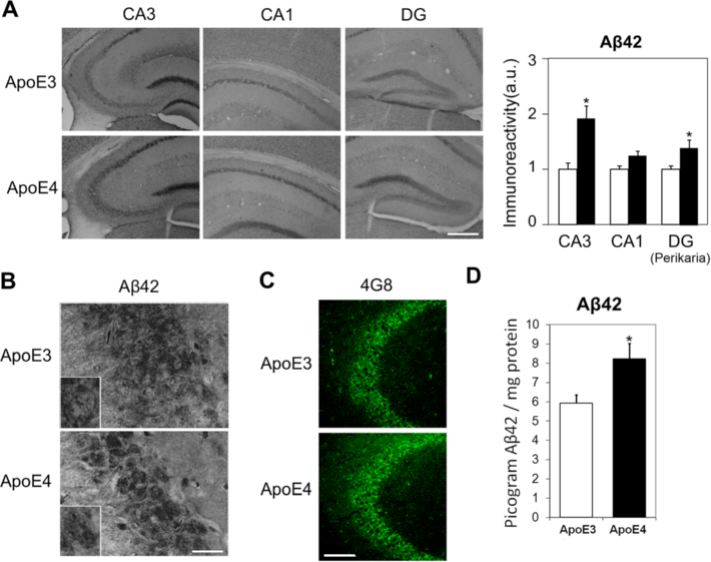
**The levels of Aβ42 in hippocampal neurons of 4-month-old apoE3 and apoE4 mice. (A)** Representative images (X10 magnification) of the indicated hippocampal fields stained with anti-Aβ42 are presented on the left. Quantification of the results (mean ± SEM; n=20 per group) of apoE3 mice (white bars) and apoE4 mice (black bars) was performed by computerized image analysis, as described in Materials and Methods, and is shown on the right. *p<0.05. Scale = 350 μ. **(B)** Representative images of CA3 at X100 magnification are shown. Scale = 30 μ. Inserts depict selected neurons at X2 magnification. **(C)** Representative confocal images (X20 magnification) of the CA3 subfield of apoE3 and apoE4 mice stained with the pan anti-Aβ Ab 4G8. Scale = 120 μ. **(D)** Aβ X-42 ELISA. Quantification of Aβ42 levels in 4-month-old apoE3 and apoE4 mice was performed utilizing an ELISA kit. Results are presented as pictograms of Aβ42 per hippocampus. *p<0.05

**Figure 5:**
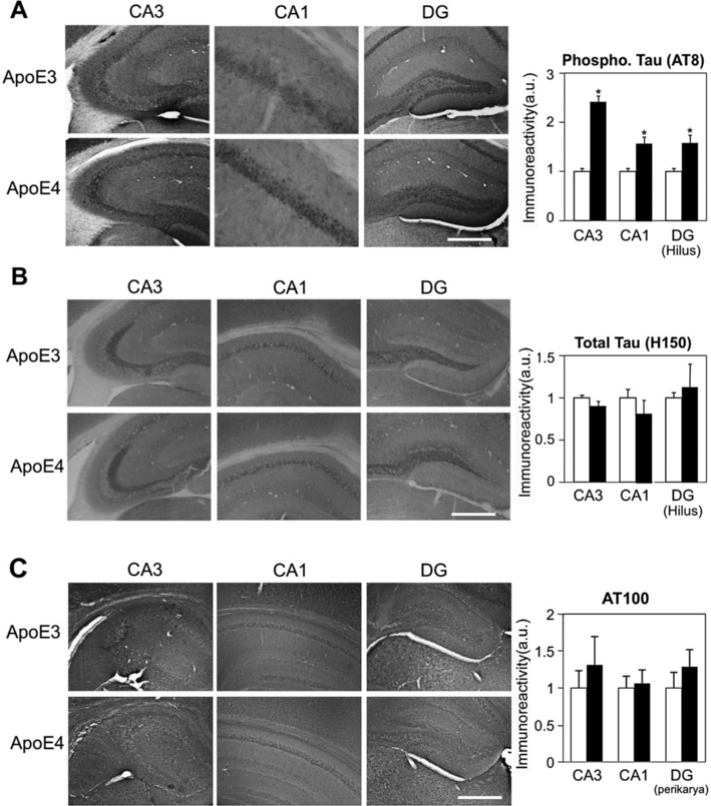
**Immunohistochemistry of tau phosphorylation and total tau levels in hippocampal neurons of 4-month-old apoE3 and apoE4 mice. (A)** Tau phosphorylation immunohistochemistry utilizing AT-8. Representative images (X10 magnification) of the indicated hippocampal subfields are presented on the left. Quantification of the results (mean ± SEM; n=20 per group) of apoE3 mice (white bars) and apoE4 mice (black bars) was performed by computerized image analysis, as described in Materials and Methods, and is shown on the right. *p<0.001. Scale = 350 μ. **(B)** Total tau levels immunohistochemistry utilizing H150. Representative images (X10 magnification) of the indicated hippocampal subfields are presented on the left. Quantification of the results (mean ± SEM; n=20 per group) of apoE3 mice (white bars) and apoE4 mice (black bars) was performed by computerized image analysis, and is shown on the right. Scale = 350 μ. **(C)** Tau phosphorylation immunohistochemistry utilizing AT100. Representative images (X10 magnification) of the indicated hippocampal subfields are presented on the left. Quantification of the results (mean ± SEM; n=6 per group) of apoE3 mice (white bars) and apoE4 mice (black bars) was performed by computerized image analysis, and is shown on the right. Scale = 350 μ.

**Figure 6:**
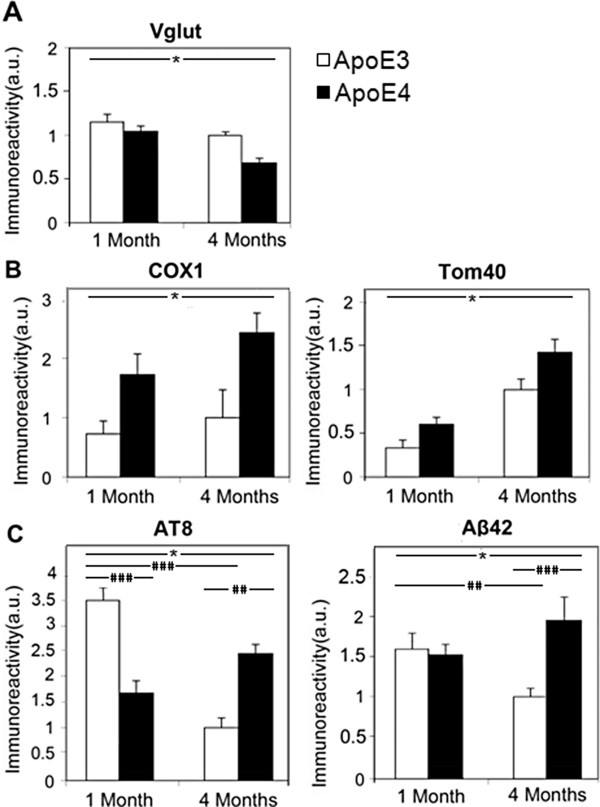
**Time course of the effects of the apoE genotype in CA3 neurons of 1- and 4-month-old apoE3 and apoE4 mice. (A)** The levels of VGlut in apoE3 (white bars) and apoE4 (black bars) mice. *p<0.02 for the effect of apoE genotype and p<0.005 for the effect of age. **(B)** The levels of the mitochondrial markers COX1 (left) and Tom40 (right) in apoE3 (white bars) and apoE4 (black bars) mice. *p<0.01 and p<0.002 of the effect for apoE genotype on Tom40 and COX1 levels, respectively, and p<0.001 for the effect of age on Tom40. **(C)** The levels of AT8 phosphorylated tau (left) and Aβ42 (right) in apoE3 (white bars) and apoE4 (black bars) mice. Two-way ANOVA revealed a significant effect for genotype×age for both the AT8 (p<0.0001) and Aβ42 (p<0.01) results. The significant post hoc comparisons of the effects of age and genotype are denoted by the hash marks as follows: ^#^p<0.05, ^##^p<0.01 and ^###^p<0.001. Results in each of the panels (n=8 mice per group) were obtained at the same time and are presented relative to the 4-month-old apoE3 group.

**Figure 7:**
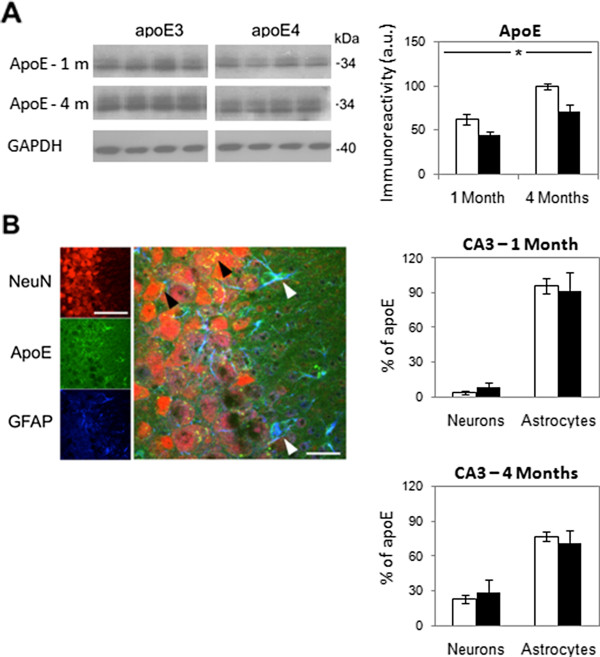
**The levels and spatial distribution of apoE in the hippocampus of 1- and 4-month-old apoE3 and apoE4 mice. (A)** ApoE immunoblots. Representative immunoblots of homogenates of whole hippocampi of 1- and 4-month-old apoE3 and apoE4 mice are presented on the left together with the GAPDH standard. Quantification of the results (mean ± SEM; n=6 per group) of apoE3 mice (white bars) and apoE4 mice (black bars) is depicted on the right. *p<0.05. **(B)** Confocal microscopy utilizing the apoE (green); neuronal marker NeuN (red); and GFAP (blue) antibodies. A representative section of the individual immunostainings and their superposition is presented. Colocalization of apoE with astrocytes is shown in light blue in the right panel (white arrowheads), whereas colocalization of apoE with neurons is shown in yellow (black arrowheads). Quantification of the relative percentage of apoE colocalized respectively with neurons and astrocytes (mean ± SEM; n=6 per group) in 1- and 4-month-old apoE3 mice (white bars) and apoE4 mice (black bars) was performed by computerized image analysis, as described in Materials and Methods, is presented on the right. Scale for left panel = 120 μ. Scale for the middle panel = 40 μ.

**Figure 8:**
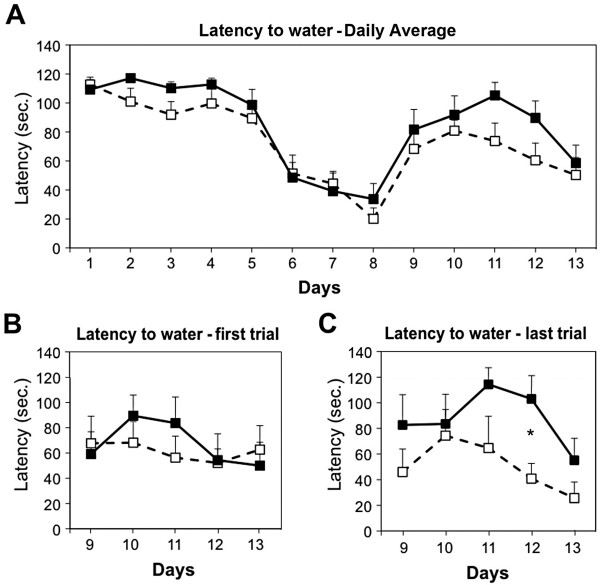
**Performance in the dry version of the Morris water maze of 4-month-old apoE3 and apoE4 mice. (A)** Average latency to the water. Daily averages (4 runs per day) of apoE3 (white squares) and apoE4 (black squares) mice (n=7 per group) for 8 days with the water-filled well in the first location and following the change in the position of the water-filled well (days 9–13). **(B)** Latency of the first run of each day (days 9–13). **(C)** Latency of the last run of each day (days 9–13). *p<0.05. ANOVA reaveled a significant effect for genotype×trial (p<0.05).

The immunohistochemistry results were obtained utilizing two sets of apoE3 and apoE4 mice, which respectively contained 8 and 12 animals for each group, except for the Tom40 experiment in which one set of mice (n=8) was employed. The results obtained with the two different cohorts were similar when analyzed separately and are presented jointly (means ± SEM) following normalization of each of the experiments to apoE3 = 100%. The immunoblot results (n=10 for all the hippocampal samples and n=5 for the CA3 samples) consisted of at least three blots and are expressed as percentages of the levels of the apoE3 mice (means ± SEM). Student's *t*-test was performed between the apoE3 and apoE4 groups (Figures 1, 2, 3, 4, 5). Bonferroni correction was employed for multiple comparisons when needed. Further analysis of interactions between genotype and age or genotype and trial (Figures 6, 7,8) were performed utilizing two-way ANOVA tests using STATISTICA software (Version 8.0 StatSoft, Inc., Tulsa, USA).

## Results

The extent to which the glutamatergic nerve terminals are affected by apoE4 at a young age was first assessed by immunohistochemical measurements of the levels of the presynaptic vesicular glutamatergic transporter 1, VGlut1 (VGlut), in 4-month-old apoE4 and apoE3-targeted replacement mice. As shown in Figure 1, staining in the CA3 and CA1 subfields was pronounced in the dendritic layers and sparse in the corresponding perikarya. Furthermore, the intensity of the VGlut staining in the dendritic layers of the CA3 and CA1 subfields was significantly lower in the apoE4 than in the corresponding apoE3 mice (i.e. a decrease of 30 ± 4% in the stratum locidum of the CA3 in the apoE4 mice p<0.0001 and of 40 ± 7.5% in the apical and basal dendritic layers of the corresponding CA1 field: p<0.001). VGlut staining in the DG, which was most pronounced in the hilus, was also lower in the apoE4 mice (a decrease of 26 ± 5.6%: p<0.001; Figure 1A). Immunoblot experiments utilizing whole hippocampus homogenates revealed, in accordance with the above immunohistochemical results, that the levels of the VGlut immunoblot band (mw = 60 kDa) were lower in the apoE4 than in the apoE3 mice (Figure 1B). It remains to be determined whether additional presynaptic and/or postsynaptic glutamatergic components are also affected by the apoE genotype.

The extent to which apoE4 affects hippocampal inhibitory GABAergic synapses was investigated utilizing the GABA synthesizing enzyme GAD67 as a marker. GAD67 resides in both the perikarya and neurites of GABA neurons [50,56]. As shown in Figure 2A, GAD67 levels in both the perikarya and the dendritic layers of CA3 were not affected by the apoE genotype. Similar results were obtained in the corresponding CA1 and DG subfields and following staining for Vgat (Vesicular GABA transporter) in all hippocampal subfields (not shown). Immunohistochemical experiments with the general synaptic vesicle marker synaptophysin revealed small apoE4-driven decreases in CA3 (15 ± 3%; p=0.005; Figure 2B), as well as in CA1 (20 ± 9%; not shown) and the DG (10 ± 3%; p=0.02; not shown). The finding that the effects of apoE4 on the general presynaptic marker synaptophysin are less robust than the corresponding effects of apoE4 on VGlut (compare Figures 1 and 2) probably reflects the differential susceptibility of different nerve types (e.g., excitatory vs. inhibitory synapses) to apoE4. Complementary measurements utilizing NeuN immunohistochemistry revealed that apoE4 did not affect the number and density of pyramidal and granular neurons in any of the hippocampal subfields (not shown).

The effects of apoE4 on the mitochondria in the hippocampus were investigated immunohistochemically and by immunoblot assays, utilizing the translocase of the outer mitochondrial membrane protein, Tom40, and the electron transport protein, COX1, as markers. The Tom40 immunohistochemistry results thus obtained are depicted in Figure 3A. As shown, the intensity of staining of the apoE4 mice increased in CA3 (42 ± 15%; p=0.04) and in the DG (88 ± 20%; p=0.004) relative to the corresponding apoE3 mice, but was not significantly affected in the CA1 subfield (an increase of 7 ± 17%). The levels of COX1 were also elevated by apoE4 (Figure 3B). This effect was specific to the CA3 subfield (an increase of 143 ± 33% relative to the apoE3 mice; p=0.03); moreover, there were no significant changes in either the CA1 (an increase of 17 ± 12%) or the DG (18 ± 11%). Higher power micrographs showed the expected punctate localization of Tom40 and COX1 in the neuronal perikarya (Figure 3C). Immunoblot assays of the CA3 subfield are depicted in Figure 3D. These experiments revealed, in accordance with the immunohistochemical results, an elevation in the Tom40 (39 ± 8%, p=0.01) and COX1 (72 ± 15%; p=0.01) levels in the apoE4 mice relative to the apoE3 mice. It remains to be determined whether these mitochondrial effects are due to direct effects of apoE4 on the mitochondria or reflect a compensatory response of the mitochondria to apoE4-induced stress.

It has been previously shown that apoE4 stimulates the accumulation of Aβ42 in hippocampal neurons following pharmacological activation of the amyloid cascade, which in turn, triggers synaptic impairments and neurodegeneration [57]. We therefore examined whether the presently observed neuronal effects of apoE4 in the young apoE4 mice are also associated with accumulation of Aβ42 in the affected neurons. As shown in Figure 4A, the perikarya of CA1 and CA3 pyramidal neurons and of the DG granular neurons stained positively for Aβ42. This was obtained utilizing the AB5078P monoclonal-Ab (mAb), whose specificity to Aβ42 has previously been confirmed [58]. In CA3 neurons the intensity of staining was significantly higher in the apoE4 than in the corresponding apoE3 mice (i.e. an increase of 98 ± 30%, p<0.0005). The levels of Aβ42 in CA1 and DG were also higher in the apoE4 mice compared with the apoE3 mice; however, these effects were smaller and less significant (increases of 29 ± 15% in CA1, p=0.06; and of 28 ± 18% in DG; p=0.04). The cellular nature of the accumulated Aβ42 was further ascertained by examining the sections at a higher magnification (Figure 4B). Similar results were obtained utilizing an ELISA kit, and total hippocampal homogenates (Figure 4D). The levels of Aβ42 in apoE4 mice were higher than in the corresponding apoE3 mice (8.23±0.79 and 5.92±0.45 picograms (pg) of Aβ42 per mg protein, respectively; p<0.05).

Control experiments revealed that the hippocampal Aβ42 staining of the apoE4 mice was significantly higher than that of a corresponding section from APP knock-out (K.O.) mice, whereas the staining of the apoE3 mice was only slightly higher than the background staining. Additional controls revealed that the patterns of staining for Aβ42 and APP (mAb 22C11) were different (see Additional file 1: Figure S1). Intracellular accumulation of Aβ42 was also observed with the pan Aβ mAb 4G8 (Figure 4C). This Ab also revealed increased staining in apoE4 than in apoE3 mice. This effect, however, was less pronounced, which is probably due to the fact that in addition to Aβ42, 4G8 also recognizes APP and other forms of Aβ.

It has been suggested that tau plays an important role in mediating the neuronal and cognitive pathological effects of apoE4 during aging [50]. The possibility that the early synaptic and pathological effects of apoE4 in young targeted replacement mice are also associated with tau-related changes was therefore examined. This was pursued by measuring the effects of apoE4 on the phosphorylation level of tau. Hippocampal sections stained with mAb AT8, which recognizes tau phosphorylated at both Ser202 and Thr205 [59], are depicted in Figure 5A. As shown, AT8 stained CA3 and CA1 pyramidal neurons as well as the granular neurons of DG and the hilus. Importantly, the intensity of AT8 staining observed in these hippocampal subfields was significantly higher in the apoE4 mice than in the apoE3 mice (i.e. an increase of 151 ± 21% in CA3, p<0.0001; an increase of 70 ± 17% in CA1; p<0.001, and an increase of 64 ± 14% in the hilus of DG; p<0.001; Figure 5A). Control experiments, utilizing the phosphorylation-insensitive tau mAb H150, revealed a staining pattern similar to that observed with AT8, but the intensities of staining were the same in the apoE3 and apoE4 mice (Figure 5B). Furthermore, the levels of the phosphorylated tau epitope, which is recognized by mAb AT100 (Thr212/Ser214) [60], were low, particularly in DG and CA3, and were similar in the apoE3 and apoE4 mice (Figure 5C). Taken together, these findings suggest that hippocampal tau of 4-month-old apoE4 mice is hyperphosphorylated and that this effect is epitope specific. Negative control experiments utilizing tau-K.O. mice revealed that the observed staining is indeed specific to tau (see Additional file 1: Figure S1). Additional experiments revealed that the extent of tau phosphorylation as well as the levels of VGlut and cellular Aβ42 in the entorhinal cortex are not affected by apoE4, suggesting that the effects of apoE4 are specific to the hippocampus.

The extent to which the effects of apoE4 on tau, Aβ42, VGlut and the mitochondria appear sequentially was assessed by measuring the effects of apoE4 on these parameters in 1-month-old mice. The results thus obtained in CA3 neurons and their comparison to the effects observed in 4-month-old mice are depicted in Figure 6 (sections from all age groups were stained together and the results obtained are normalized relative to apoE3 at 4 months, whose value was set at 1). Two-way ANOVA on the VGlut results (Figure 6A) revealed a significant effect for apoE genotype (p<0.02) and age (p<0.005) and a non-significant effect for genotype×age (p=0.25). This suggests that the levels of VGlut are lower in the apoE4 than in the apoE3 mice and that they both decrease similarly over time. The results thus obtained with the mitochondrial markers Tom40 and COX1 are depicted in Figure 6B. Two-way ANOVA of the Tom40 results (Figure 6B) revealed a significant effect for apoE genotype (p<0.01) and age (p<0.001) and that the age dependency of the Tom40 levels of the apoE4 and apoE3 mice were similar (p=0.5 for genotype×age). The COX1 levels of apoE4 mice (Figure 6A) were also higher than those of the apoE3 mice (p<0.005 for the effect of apoE genotype). It followed the same pattern as that obtained with Tom40 except that in the case of COX1 the increase with age was not statistically significant. Taken together, these findings suggest that both age and apoE4 independently cause a decrease in the levels of VGlut and increase in the levels of the mitochondrial markers.

The effects of apoE genotype and age on Aβ42 levels are depicted in Figure 6C. Two-way ANOVA of these results revealed a significant effect for genotype×age (p<0.001). Further post hoc analysis revealed that the levels of Aβ42 at 1 month in the apoE3 and apoE4 mice were similar and that they decreased significantly with time in the apoE3 mice and insignificantly increased in the corresponding apoE4 mice. This yielded a significant difference at 4 months between the Aβ42 levels of the apoE4 and apoE3 mice (p<0.01).

The age dependency of tau phosphorylation in CA3 neurons of the apoE3 and apoE4 mice is depicted in Figure 6C. Two-way ANOVA of these results revealed a significant effect for genotype×age (p<0.001). This was associated with significantly elevated levels of phosphorylated tau in the 1-month-old apoE3 mice relative to the corresponding apoE4 mice (p<0.001) and with a significant age dependent reduction in the levels of tau phosphorylation in the apoE3 mice (p<0.001). In contrast, the levels of tau phosphorylation in the apoE4 mice increased between 1 and 4 months of age, however this effect was not statistically significant.

Thus, the pattern obtained is biphasic: at 1 month, tau is hyperphosphorylated in the apoE3 relative to the apoE4 mice, whereas at 4 months the phosphorylation levels of the apoE3 mice decrease and are consequently significantly lower than those of the corresponding apoE4 mice. The putative mechanisms that may underlie this biphasic effect are presented in the discussion. However, regardless of the mechanisms involved, these findings show that the effects of the apoE genotype, which are reflected by tau phosphorylation, also start at 1 month or before.

Taken together, these results define a time window for the effects of apoE4 on CA3 neurons that occur at 1 month or before and are reflected by changes in tau phosphorylation and the mitochondrial parameters. This is then followed by presynaptic pathology and the accumulation of neuronal Aβ42. Similar age-dependent patterns were observed in CA1 and DG, where the tau and mitochondrial changes preceded the decrease in VGlut (not shown).

Measuring the effect of apoE4 on the apoE levels in the hippocampus of 4-month-old mice revealed, in accordance with a previous reports [61,62] that they were lower in the apoE4 than in the apoE3 mice (Figure 7A). Similar results were obtained with 1-month-old mice, whose apoE levels were, however, lower than those of the corresponding 4-month-old mice. Confocal microscopy colocalization experiments revealed that in 1-month-old mice most of the apoE was colocalized with astrocytes, whereas in the 4-month-old mice, the fraction of apoE associated with neuronal perikarya increased (Figure 7B). Importantly, the relative distribution of apoE4 in these compartments was not affected by the apoE genotype (Figure 7B).

The effects of apoE4 on the cognitive performance of young apoE4 mice were assessed utilizing a dry version of the Morris Swim test in which water-deprived mice are tested for their ability to learn the location of a water-filled well. As shown in Figure 8, both the apoE3 and apoE4 mice learned the position of the water-filled well and shortened their latencies to this well such that they reached a plateau of about 30 sec by days 6–8 (see days 1 to 8 in Figure 8A). The water-filled well was then moved to a new position and the mice were tested for 5 additional days (days 9–13 in Figure 8). As shown, the performance of both groups deteriorated in the first 2 days after the position of the water-filled well was changed. Following the deterioration, which was similar in apoE3 and apoE4 mice, the performance of the mice improved and this effect seemed to occur more rapidly in apoE3 than in apoE4 mice. This trend was not statistically significant (repeated-measures ANOVA p=0.18 and on the 4th day p=0.1). However, a separate analysis of the results obtained on the first and last of the 4 daily runs during days 9–13 revealed significant differences. As shown in Figure 8C, the performance of the apoE4 mice in the last run of each day was significantly impaired (repeated-measures ANOVA p=0.02 and on the 4th day p=0.01). Conversely, no differences between the apoE3 and apoE4 mice were observed in the first run of each day (repeated-measures ANOVA p=0.73 and on the 4th day p=0.92; Figure 8B).

Further two-way ANOVA analysis of the effect for apoE genotype and for trials revealed that genotype×trial (p=0.02) had an effect (p<=0.02), thus confirming that the effects of apoE4 are trial dependent. The performance in the first of the daily runs is related to long-term memory, whereas the performance in the last daily run is related to short-term memory [63]. This suggests that the cognitive deficit of the young apoE4 mice is related to impaired short-term working memory.

## Discussion

This study investigated the extent to which the early pathological effects of apoE4, known to occur in man, also occur in young apoE4-expressing mice. This revealed that the levels of the presynaptic glutamatergic transporter VGlut are lower in CA3, CA1, and DG hippocampal neurons of 4-month-old apoE4-targeted replacement mice than in the corresponding apoE3 mice. In contrast, the corresponding inhibitory GABAergic nerve terminals and perikarya were not affected by apoE genotype. This synaptic effect was associated with hyperphosphorylated tau in these hippocampal subfields and with the accumulation of Aβ42 in CA3 neurons. Further experiments revealed that the mitochondrial markers Tom40 and COX1 were also elevated by apoE4, and that the levels of Tom40, but not COX1, were elevated in the DG. A summary of these findings is presented in Table 1. Time-course studies revealed that the apoE4-driven accumulation of Aβ42 and the associated decrease in VGlut develop after the age of 1 month and that they are preceded by mitochondrial and tau phosphorylation apoE-genotype specific effects.

**Table 1:** Summary of the effects of apoE4 on the hippocampus

**Area parameter**	**CA3**		**CA1**		**DG**	
	**Perikarya**	**Neurites**	**Perikarya**	**Neurites**	**Perikarya**	**Neurites**
VGlut		−30±5		−40±8		−26±6
(% decrease)		P<0.0001		P<0.001		P<0.001
GAD67	+14±6	+14±7	+5±10		+9±4	
(% increase)	N.S.	N.S.	N.S.		N.S.	
Tom40	+42±15		+7±17		+88±20	
(% increase)	P=0.04		N.S.		P=0.004	
COX1	+143±33		+17±12		+18±11	
(% increase)	P=0.03		N.S.		N.S.	
Tau (AT8)	+150±21		+70±17			+65±14
(% increase)	P<0.0001		P<0.001			P<0.001
Aβ42	+95±20		+20±8		+34±13	
(% increase)	P<0.001		N.S.		P=0.04	

N.S. = Not Significant.

The present finding that glutamatergic neurons are negatively affected by apoE4 is in accordance with previous electrophysiological and anatomical observations with targeted replacement apoE4 mice and other models showing that apoE4 impairs glutamatergic synapses and neuronal transmission [64-67]. Previous studies with older apoE4 mice revealed that GABAergic neurons are affected by apoE4 and that this effect develops with age and is robust in 10-12-month-old mice [50]. Taken together, the results suggest that glutamatergic neurons are an early target of apoE4 and that this effect is followed by subsequent GABAergic pathology. The finding that other components of the glutamatergic synapse, such as synaptic spines [65], postsynaptic glutamatergic receptors [64,68], and the scaffold protein PSD-95 [69] are decreased by apoE4 suggests that the presently observed apoE4-driven decrease in VGlut is not specific to this molecule and is associated with impaired glutamatergic function. It is important to note that the magnitude and direction of the effects of apoE4 are affected by diet. Accordingly, unlike presently observed, the levels of VGlut are elevated by apoE4 in mice fed a DHA-depleted diet but were the same in apoE3 and apoE4 mice that were fed a high-DHA diet [54]. The mechanisms underlying the effects of lipids on the balance between the presently observed apoE4-driven reduction in VGlut levels and the effect observed in DHA-depleted apoE4 mice remain to be determined. However, since apoE4-driven synaptic loss seems to be accompanied by an increase in synaptic area [70], it is possible that the overall effect of apoE4 on VGlut, and its polarity reflect the extent to which diet affects these processes. Further sub-cellular fractionation studies are required in order to unravel the specifics of the mechanism underlying the effect of apoE4 on the life cycle of VGlut.

The present finding that the mitochondria are affected by apoE4 in young mice is in accordance with previous findings, such as reduced COX1 activity in the brains of young adult apoE4 carriers [46], a genetic association between apoE4 and the TOMM40 gene [48,71], and the *in vitro* effects of apoE4 on mitochondrial activity [47,72]. However, since the presently observed mito-chondria-related effects of apoE4 are up-regulation of the levels of the mitochondrial proteins COX1 and Tom40, it is possible that this effect represents a compensatory defense response to the related pathological effects of apoE4. Accordingly, the observed elevation in COX1 and Tom40 levels, which is already apparent in the CA3 neurons at the age of 1 month, may reflect activation of a defense mechanism, which at 1 month, but not at 4 months, is able to counteract the effect of apoE4 on Aβ and VGlut. It is, however, possible that the observed up-regulation of the mitochondrial proteins reflects an apoE4-dependent functional mitochondrial abnormality. The effects of apoE4 on the mitochondria are neuron specific and occur mainly in CA3 neurons. It remains to be determined whether this is due to specific properties of the mitochondria of the CA3 neurons, which render them more responsive to stressful stimuli, or to increased susceptibility of the CA3 neurons to apoE4. The finding that the levels of Aβ42 and phosphorylated tau are also highest in CA3 neurons is consistent with both of these interpretations.

The present finding that apoE4 increases the accumulation of neuronal Aβ42 and hyperphosphorylated tau in hippocampal neurons is in agreement with previous observations. However, these studies rely on mechanistic hypothesis-driven models, such as APP and apoE4 double transgenes [73], pharmacological activation of the amyloid cascade for Aβ [57], and transgenic mice that express tau in neurons [43,44,74]. The novelty of the present observations is that the apoE4-driven accumulation of neuronal Aβ42 and hyperphosphorylated tau occurs spontaneously in the absence of any tau or Aβ-related manipulations.

These findings raise important questions regarding the mechanisms by which apoE4 triggers the accumulation of Aβ42 and hyperphosphorylated tau in hippocampal neurons and the possible role of these molecules in mediating the synaptic pathological effects of apoE4. Previous studies have shown that apoE4 can enhance the effects of Aβ by several mechanisms. These include decreased degradation and clearance of human Aβ42 from the brain [75-78], as well as stimulation of the intraneuronal accumulation of mouse Aβ [32]. The fact that the apoE4-driven accumulation of Aβ42 is neuron specific and is highest in CA3 neurons argues against a general hippocampal mechanism and favors a CA3-based neuron-specific mechanism. The intraneuronal accumulation of Aβ can be driven by apoE receptors [79] whose levels are affected by apoE genotype. Aβ is localized to glutamatergic synapses [80,81] and can decrease synaptic activity [82]. It is thus possible that Aβ42 plays a role in the observed synaptic pathology of the CA3 neurons via such a mechanism. However, since the loss of VGlut in the CA1 and DG, which is similar to that observed in CA3 (Figure 1), is associated with only a small increase in neuronal Aβ42 (Figure 4), additional non-Aβ42-driven mechanisms may also be involved.

ApoE4 can affect tau phosphorylation either directly by binding to tau [83], or via apoE receptors and downstream signaling, which can affect kinases such as GSK3β [84-86]. The finding that at 1 month, unlike at 4 months, tau is more phosphorylated in the apoE3 than in the apoE4 mice and that this age-dependent effect is due to a specific decrease in tau phosphorylation between 1 and 4 months in the apoE3, with no change in the apoE4 mice, suggests that a mechanism responsible for tau phosphorylation and subsequent dephosphorylation is missing in the apoE4 mice. Tau is transiently hyperphosphorylated at the AT8 epitopes (202/205) and numerous other sites during neuronal development [87-89]. It is thus possible that the decreased AT8 phosphorylation in 1-month-old mice (see Figure 5) reflects developmental effects of apoE4. Additional studies starting at younger ages and utilizing embryos are required in order to further characterize this effect of apoE on tau phosphorylation and for identifying putative kinases and phosphatases that may play a role in mediating the isoform-specific effects of apoE on tau phosphorylation. Tau hyperphosphorylation can have numerous pathological effects including depolymerization of microtubules and subsequent impairments of axonal transport [90], as well as the formation of cytotoxic tau aggregates [91]. Since the excess of 202/205 tau phosphorylation and the reduced VGlut levels are apparent in CA3, CA1, and DG neurons, it is possible that such tau-related mechanisms may mediate the effects of apoE4 on the glutamatergic nerve terminals.

The molecular mechanism underlying the presently observed effects of apoE4 on VGlut, Aβ42, AT8 tau phosphorylation as well as the mitochondrial parameters are not known. Preliminary findings suggest that the levels of the apoE receptor apoER2 in the CA3, CA1, and DG hippocampal neurons are markedly reduced in the apoE4 mice (to be published). This is in accordance with previous observations [64] and suggests that the observed effects of apoE4 may be mediated by impaired apoER2 signaling. It remains to be determined whether these effects are triggered via a loss of function mechanism (e.g., apoE4 < apoE3. see Figure 7), or via a gain of toxic function mechanism.

The present finding that 4-month-old apoE4 mice are cognitively impaired in dry maze is in accordance with the recent finding that the learning and memory performances of young apoE4 mice in the fear conditioning paradigm is also impaired [92].

It has recently been shown that the performance of rats in a spatial navigation test across days reflects the efficacy of reference memory, whereas the corresponding performance within a testing session is a measure of working memory [63]. Accordingly, the present finding that the performance of the apoE4 mice is impaired in the last but not the first daily run following the change in position of the water-filled well (Figures 6B and 6C) suggests that the working memory of the apoE4 mice is impaired. It is, however, possible that additional behavioral features such as motivation or susceptibility to stress also affect the performance of the apoE4 mice.

The neurochemical findings that the isoform-specific effects of apoE4 on tau phosphorylation and on the mitochondrial parameters are already apparent at the age of 1 month, whereas the associated accumulation of Aβ and glutamatergic pathology evolve later, suggest that tau phosphorylation and the mitochondrial changes reflect early apoE4-driven processes that are followed by the Aβ and synaptic changes. These processes are particularly robust in CA3 neurons. The causal relationship between the different neurochemical effects of apoE4 and the extent to which they mediate the behavioral effects of apoE4 remain to be determined.

The extent to which the observed effects of apoE4 are mediated by either gain or loss of function is not known. We have recently shown that the pathological synergistic interactions between apoE4 and Aβ are more pronounced in apoE4 than in apoE-K.O. mice, suggesting that the interaction between apoE4 and Aβ is mediated via a gain of toxicity mechanism [93]. However, since the levels of apoE are lower in the apoE4 than in the apoE3 mice, we cannot rule out the possibility that a loss of function mechanism also plays a role in mediating the effects of apoE4.

Recent *in vivo* and *in vitro* studies revealed that apoE4 impairs the blood brain barrier (BBB) [94,95]. Since these effects are already apparent at a very young age in apoE4-targeted replacement mice [95], it is possible that impairments in the BBB play a role in initiating the effects of apoE4 on Aβ, tau, and VGlut. However, since the effects presented are neuron specific (see Figures 1, 3, and 4), additional neuronal mechanisms, downstream to the BBB, must also play a role.

Gene expression studies of AD brains revealed that apoE4 is associated with altered transcription of multiple gene transcripts including the down-regulation of genes related to synaptic plasticity and function [42,96]. Recent studies suggest that in addition to the effects of apoE4 on brains of the aged population [97,98], it also affects the brains of apparently healthy younger apoE4 carriers [8,31,46,99-101]. Furthermore, it has been recently shown that the human brains of neonates are also affected by apoE4 [102]. Accordingly, it is possible that the effects of apoE4, which are already apparent in the developing brain at a young age, may play a role in the subsequent induction of the disease later in life. The present study, which focuses on brain neurons in young apoE4 mice, and recent complementary reports that focused on the vasculature [69,94] and glia [69,94] of these mice, are consistent with this hypothesis, and suggest that the pathological effects of apoE4 start much earlier in life than previously thought.

Another important implication of these findings is that young apoE4 mice provide an unbiased model for studying the mechanisms underlying the pathological effects of apoE4 in the absence of any mechanism-driven manipulations. However, the jury is still out regarding the cellular and molecular mechanisms that mediate the effects of apoE4 *in vivo* and whether they are due to gain of toxic function and/or to a loss of function. The present model, combined with the recently described pharmacological manipulations that elevate the total level of brain apoE [103] and of mAbs that are directed specifically at apoE4 [104], now provide the means to address these important issues.

In conclusion, the present findings show that the pathological effects of apoE4 in targeted replacement mice are already apparent in young 4-month-old mice and that at this stage the glutamatergic system is particularly susceptible to apoE4. These effects are associated with the accumulation of neuronal Aβ42, hyperphosphorylated tau, and an increase in mitochondrial markers. This suggests that the pathological effects of apoE4 are already apparent at a very young age and that Aβ42, tau, and the mitochondria play a role in mediating the observed early apoE4-driven synaptic pathology. Young apoE4 mice thus provide an unbiased and hypothesis-independent model for studying the early pathological effects of apoE4.

## Competing interests

The authors declare that they have no competing interests.

## Authors’ contributions

OL* carried out the biochemical studies with the VGlut, GAD67, synaptophysin, tau and Aβ markers, as well as preformed the double-labeled staining for apoE, NeuN and GFAP and the behavioral tests and also drafted the manuscript. ABC* carried out the biochemical studies with the COX1, Tom40 and apoE markers and drafted the manuscript. *The authors contributed equally to this manuscript. DMM conceived the study, and participated in its design, coordination of the investigation, and helped to edit the manuscript for consideration for publication. All authors read and approved the final manuscript.

## Supplementary Material

Additional file 1: Figure S1 Legend: Negative controls of the AT8 tau and Aβ42 immunohistochemical staining. (A) AT8 staining of hippocampal sections of tau-K.O. (Jackson #007251) and WT mice showing that staining is absent in the tau KO mice. Scale = 300 μ. (B) Aβ42 staining of hippocampal sections of APP-K.O. mice and WT mice showing that staining is absent in the APP-K.O. mice. The APP-K.O mice were kindly provided by Prof. H. Muller. Scale = 300 μ. (B) Aβ42 staining of hippocampal sections of APP-K.O. mice and WT mice showing that staining is absent in the tau KO mice. Scale = 300 μ. (C) Representative image of hippocampal CA3 neurons of apoE4 mice co-stained for Aβ42 and APP (mAb directed against N-teminal APP, 22C11). As can be seen, the patterns of staining of the 2 Abs are different (less than 15% of Aβ42 is colocalized to APP). Similar results were obtained with corresponding sections from apoE3 mice. Scale = 30 μ. Click here for file
