# Supplementary material for: ApoE4 induces Aβ42, tau, and neuronal pathology in the hippocampus of young targeted replacement apoE4 mice
Source: Mol Neurodegener. 2013 May 17;8:16. doi: 10.1186/1750-1326-8-16 (PMC3659080; doi:10.1186/1750-1326-8-16)
Supplement: Additional file 1: Figure S1 — Legend: Negative controls of the AT8 tau and Aβ42 immunohistochemical staining. (A) AT8 staining of hippocampal sections of tau-K.O. (Jackson #007251) and WT mice showing that staining is absent in the tau KO mice. Scale = 300 μ. (B) Aβ42 staining of hippocampal sections of APP-K.O. mice and WT mice showing that staining is absent in the APP-K.O. mice. The APP-K.O mice were kindly provided by Prof. H. Muller. Scale = 300 μ. (B) Aβ42 staining of hippocampal sections of APP-K.O. mice and WT mice showing that staining is absent in the tau KO mice. Scale = 300 μ. (C) Representative image of hippocampal CA3 neurons of apoE4 mice co-stained for Aβ42 and APP (mAb directed against N-teminal APP, 22C11). As can be seen, the patterns of staining of the 2 Abs are different (less than 15% of Aβ42 is colocalized to APP). Similar results were obtained with corresponding sections from apoE3 mice. Scale = 30 μ. [file 1750-1326-8-16-S1.doc]

**Table 1:** Summary of the effects of apoE4 on the hippocampus

| area  Parameter | CA3 | | CA1 | | DG | |
| --- | --- | --- | --- | --- | --- | --- |
|  | Perikarya | Neurites | Perikarya | Neurites | Perikarya | Neurites |
| VGlut  (% decrease) |  | -30±5  P<0.0001 |  | -40±8  P<0.001 |  | -26±6  P<0.001 |
| GAD67  (% increase) | +14±6  N.S. | +14±7  N.S. | +5±10  N.S. |  | +9±4  N.S. |  |
| Tom40  (% increase) | +42±15  P=0.04 |  | +7±17  N.S. |  | +88±20  P=0.004 |  |
| COX1  (% increase) | +143±33  P=0.03 |  | +17±12  N.S. |  | +18±11  N.S. |  |
| Tau (AT8)  (% increase) | +150±21  P<0.0001 |  | +70±17  P<0.001 |  |  | +65±14  P<0.001 |
| Aβ42  (% increase) | +95±20  P<0.001 |  | +20±8  N.S. |  | +34±13  P=0.04 |  |

N.S. = Not Significant
